# Relapse of congenital thrombotic thrombocytopenic purpura, after spontaneous remission, in a second-trimester primigravida: case report and review of the literature: Recidiva, após remissão espontânea, de púrpura trombocitopênica trombótica congênita em primigesta no segundo trimestre de gestação: relato de caso e revisão da literatura

**DOI:** 10.1590/1516-3180.2016.0188201116

**Published:** 2017-04-20

**Authors:** Donavan de Souza Lúcio, Jacqueline Foelkel Pignatari, Marcelo Gil Cliquet, Henri Augusto Korkes

**Affiliations:** I MD. Family Medicine Resident, Municipal Health Department, Prefeitura Municipal de Florianópolis (PMF-SC), Florianópolis (SC), Brazil.; II MD. Internal Medicine Resident, Department of Internal Medicine, Faculdade de Ciências Médicas e da Saúde (FCMS), Pontifícia Universidade Católica de São Paulo (PUC-SP), São Paulo (SP), Brazil.; III MD, MSc, PhD. Chairman, Department of Hematology, Faculdade de Ciências Médicas e da Saúde (FCMS), Pontifícia Universidade Católica de São Paulo (PUC-SP), São Paulo (SP), Brazil.; IV MD, MSc. Attending Physician, Department of Obstetrics and Gynecology, Faculdade de Ciências Médicas e da Saúde (FCMS), Pontifícia Universidade Católica de São Paulo (PUC-SP), São Paulo (SP), Brazil.

**Keywords:** Purpura, thrombotic thrombocytopenic, Anemia, hemolytic, Pregnancy, high-risk, Stillbirth, ADAM proteins

## Abstract

**CONTEXT:**

Thrombotic microangiopathy syndrome or thrombotic thrombocytopenic purpura-hemolytic uremic syndrome (TTP-HUS) describes distinct diseases sharing common pathological features: microangiopathic hemolytic anemia and thrombocytopenia, without any other apparent cause.

**CASE REPORT:**

An 18-year-old second-trimester primigravida presented with a history of fifteen days of intense weakness, followed by diarrhea over the past six days. She reported having had low platelets since childhood, but said that she had never had bleeding or menstrual abnormalities. Laboratory investigation showed anemia with schistocytes, thrombocytopenia and hypohaptoglobulinemia. Red blood cell concentrate and platelet transfusions were performed. The hypothesis of TTP or HUS was put forward and ADAMTS13 enzyme activity was investigated. The patient evolved with increasing platelet counts, even without specific treatment, and she was discharged. One month afterwards, she returned presenting weakness and swollen face and legs, which had developed one day earlier. The ADAMTS13 activity was less than 5%, without presence of autoantibodies. Regarding the two previous admissions (at 9 and 16 years of age), with similar clinical features, there was spontaneous remission on the first occasion and, on the second, the diagnosis of TTP was suspected and plasmapheresis was performed, but ADAMTS13 activity was not investigated.

**CONCLUSION:**

To date, this is the only report of congenital TTP with two spontaneous remissions in the literature This report reveals the importance of suspicion of this condition in the presence of microangiopathic hemolytic anemia and thrombocytopenia without any other apparent cause.

## RESUMO

**CONTEXTO:**

A síndrome de microangiopatia trombótica, ou púrpura trombocitopênica trombótica-síndrome hemolítico urêmica (PTT-SHU), descreve doenças diversas com clínica e achados patológicos comuns: anemia hemolítica microangiopática e trombocitopenia, na ausência de outra causa aparente.

**RELATO DO CASO:**

Primigesta de 18 anos no segundo trimestre apresenta-se com quadro de 15 dias de fraqueza intensa seguida por diarreia há seis dias. Relata ter plaquetas baixas desde a infância e nega sangramentos e anormalidades menstruais. Investigação laboratorial identificou anemia com esquizócitos, plaquetopenia e hipo-haptoglobulinemia. Foi realizada transfusão de plaquetas e concentrado de hemácias. A hipótese de PTT ou SHU foi aventada e realizou-se pesquisa da atividade da enzima ADAMTS13. A paciente evoluiu com elevação das plaquetas, mesmo sem tratamento específico, tendo alta. Retornou após um mês da alta com queixa de fraqueza há um dia e inchaço de face e pernas. A atividade da ADAMTS13 foi menor que 5%, sem autoanticorpos. Nas duas internações anteriores (aos 9 e 16 anos), com quadros similares, houve remissão espontânea na primeira internação e, na segunda, o diagnóstico de PTT foi suspeitado e foi realizada plasmaférese, porém sem a pesquisa da atividade da ADAMTS13.

**CONCLUSÃO:**

Até esta data, este é único relato de TTP congênita com duas remissões espontâneas na literatura. Este relato revela a importância da suspeição desta patologia na presença de anemia hemolítica microangiopática e trombocitopenia sem outra causa aparente.

## INTRODUCTION

Thrombotic microangiopathy syndrome (TMS) or thrombotic thrombocytopenic purpura-hemolytic uremic syndrome (TTP-HUS) describes distinct diseases sharing common pathological features: microangiopathic hemolytic anemia (defined by the presence of schistocytes in blood smears) and thrombocytopenia (with or without neurological or renal abnormalities) without any other apparent cause.^1^^,^^2^^,^^3^

The variety of presentations and lack of specific diagnostic criteria for TTP-HUS hinder and delay its recognition and treatment by means of plasmapheresis.^1^ Children with microangiopathic hemolytic anemia, acute renal failure and thrombocytopenia have been classified as presenting hemolytic uremic syndrome (HUS). This disease is typically preceded by diarrhea and abdominal pain, caused by Shiga toxins that are produced by bacteria such as *Escherichia coli* 0157:H7. It has low mortality, and 91% of typical HUS children survive without plasmapheresis, thus suggesting that TTP and HUS are two different syndromes.^1^ However, their diagnostic criteria are the same, and although renal failure and neurological abnormalities are characteristic of HUS and TTP, respectively, these features may never occur.^1^

Several conditions, such as infections, surgery and pregnancy can precipitate TMS.^3^ Congenital TTP is the most frequent manifestation of TMS during pregnancy.^3^^,^^4^ This is a rare disorder with only just over 100 case descriptions worldwide.^5^ It is caused by mutations in the ADAMTS13 enzyme (“a disintegrin and metalloprotease with a thrombospondin type 1 motif, member 13”). This is a protease that cleaves von Willebrand factor, which is a multimer synthesized by endothelium that, if degradation does not occur, accumulates and thus leads to spontaneous formation of microthrombi in the microcirculation.^6^^,^^7^ Congenital TTP is characterized by low ADAMTS13 activity in the absence of anti-ADAMTS13 autoantibodies, which are present in acquired TTP.^8^

The initial evaluation should investigate possible secondary causes such as pregnancy, autoimmune disease and infection by the human immunodeficiency virus. It needs to rule out other causes of microangiopathy such as neoplasia and disseminated intravascular coagulation.^5^ Presence of a family history may suggest congenital cases.^5^ Its main differential diagnoses during pregnancy include disseminated intravascular coagulation and the HELLP syndrome (hemolysis, elevated liver-enzyme levels and low platelets).^9^

We searched the MEDLINE (via PubMed), LILACS (via BVS) and UpToDate databases for the terms: “Congenital Thrombotic Thrombocytopenic Purpura”, “Hereditary Thrombotic Thrombocytopenic Purpura”, “Familial Thrombotic Thrombocytopenic Purpura”, “Upshaw-Schulman syndrome”, “Thrombotic Thrombocytopenic Purpura”, “Microangiopathic Hemolytic Anemia”, “Pregnancy” and “ADAMTS13” (Table 1). We found 43 cases of congenital thrombotic thrombocytopenic purpura in pregnant women reported in the literature.

**Table 1.**
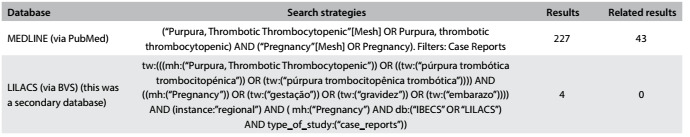
Summary of search strategies conducted on January 15, 2017

### Case report

An 18-year-old primigravida, with a fetus of 16 weeks of gestational age, presented to the obstetrics emergency department with a condition of intense weakness, followed by diarrhea, that she had had for 15 days. She said that she did not have any fever, headache, bleeding, seizures, neurological abnormalities, urinary abnormalities, rash or shortness of breath, and that she was not using any medications.

She reported that she had had a condition of “low platelets” since childhood (but without any bleeding and with regular menstrual cycle and flow), and that she had been admitted to this hospital (without remembering any details), where she was followed up by the hematology and nephrology departments, with discharge from the hospital three years before the present case.

In the medical files relating to this patient, we found two previous admissions to the emergency department of this hospital. At the first admission, when she was nine years old, she presented with acute hemolysis, uremia and ascites, which progressed to urosepsis and seizures. She was then kept in hospital for treatment with steroids, antibiotic therapy and peritoneal dialysis. Subsequently, she was followed up as an outpatient by the hematology department and she evolved with spontaneous and sustained normalization of platelets and hemoglobin, upon which she was discharged with orientations.

At the second admission, she was 16 years old and presented with abdominal pain, vomiting and hematuria. In the investigation, Coombs-negative hemolytic anemia with thrombocytopenia and elevated blood urea nitrogen were found. An abdominal ultrasound showed mild alteration of renal texture. She was kept in hospital to receive a methylprednisolone pulse because of the suspicion of rapidly progressive glomerulonephritis. Presence of schistocytes in blood smears suggested a diagnosis of TTP or HUS. Plasmapheresis was performed and steroids were administered, without further investigations. Because her condition evolved with clinical and laboratory improvement, she was discharged with a prescription for prednisone 40 mg/day and was given generic follow-up orientations.

On physical examination in the present case, she had an axillary temperature of 37.9 °C (100.22 °F), oxygen saturation of 96% in room air, blood pressure of 110 x 80 mmHg, slight jaundice and pallor. Fetal heartbeats were present and normal.

Laboratory tests showed hemoglobin of 4.2 g/dl, 13,000 platelets/mm³, hematuria, lactate dehydrogenase (LDH) of 2,014 U/l, erythrocyte sedimentation rate (ESR) of 135 mm/h, and C-reactive protein (CRP) of 62.4 mg/l. Prothrombin and activated thromboplastin time were normal. Thus, we ordered the tests summarized in Table 2. This investigation showed presence of anemia with reticulocytosis, schistocytes in blood smears, hypohaptoglobulinemia, left-shift leukocytosis, secondary iron overload due to hemolysis and elevation of D-dimer and antiphospholipid antibodies (Table 2).

**Table 2.**
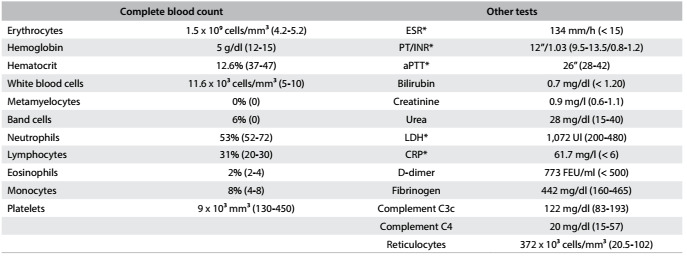
Laboratory tests ordered on the second day after admission and results

*ESR = erythrocyte sedimentation rate; PT/INR = prothrombin time/international normalized ratio; aPTT = activated partial thromboplastin time; LDH = lactate dehydrogenase; CRP = C-reactive protein.

Red blood cell and platelet transfusions were performed. The hypothesis of TTP or HUS was suggested and we requested an analysis on ADAMTS13 activity. Increasing platelet levels and normalization of hemoglobin were observed, even without specific treatment. The patient was instructed to continue treatments as an outpatient of the nephrology department. Evaluation of prothrombin and activated thromboplastin time was requested and both of these were normal.

One month after having been discharged, she returned to this hospital complaining of weakness, with swollen face and legs that had developed one day earlier. She was in a good general condition, with presence of normal fetal heartbeats. At this new admission, she had the same laboratory abnormalities, but this time her creatinine was 1.6 mg/dl. A hematological assessment was requested: the results from the ADAMTS13 activity analysis had just been received. The analysis showed that the activity level was less than 5%, without any presence of anti-ADAMTS13 autoantibodies. After this confirmation of the presence of congenital TTP, plasmapheresis was scheduled and plasma infusion was implemented at a dose of 10 ml/kg every 8 hours while waiting for the plasmapheresis.

However, plasmapheresis was delayed because of the high risk of bleeding. During this period, she developed paresthesia in the face and limbs and became fatigued. The fetal heartbeats became inaudible and fetal death was confirmed by means of ultrasound. Delivery was induced using misoprostol, which led to expulsion of the conceptus after 15 hours. Histopathological examination on the fetus showed that it weighed 318 grams and had a gestational age compatible with 16-17 weeks of pregnancy, with a second-trimester hyalinized placenta.

The feasibility of plasmapheresis was confirmed four days after confirmation of the diagnosis and indication of the procedure. Plasma exchange was performed using 16 fresh frozen plasma units, with a good response, and the patient was returned to a plasma infusion regimen two days later. Because she responded to maintenance therapy, we then discharged her for outpatient treatment.

## DISCUSSION

Through descriptions of TTP cases, reviewed in 1966, the clinical pentad formed by anemia, thrombocytopenia, fever and neurological and kidney disorders became the diagnostic criteria for TTP.^10^ Nowadays, with the availability of plasmapheresis, only thrombocytopenia and microangiopathic hemolytic anemia are necessary for suspicion of TTP and for implementation of early treatment.^11^ After the first case report of TTP associated with pregnancy, subsequent descriptions found the same association, first manifested through gastrointestinal symptoms and then through hypertension closer to term, with serious neurological and renal abnormalities. These cases evolved to death through disseminated intravascular coagulation, in the absence of plasmapheresis.^12^ The major features of acquired TTP, congenital TTP and hemolytic uremic syndrome are summarized in Table 3.

**Table 3.**
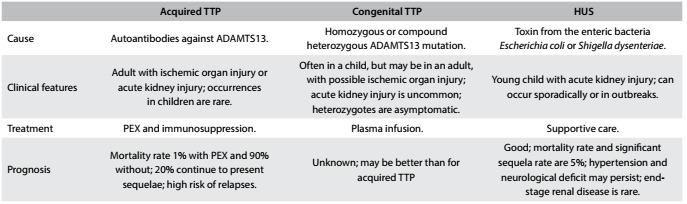
Major features of TTP-HUS^2^^,^^3^^,^^5^

TTP = thrombotic thrombocytopenic purpura; HUS = hemolytic uremic syndrome; ADAMTS13 = “a disintegrin and metalloprotease with a thrombospondin type 1 motif, member 13”; PEX = plasma exchange.

The incidence of TTP is 1 in 25,000 to 1 in 198,000 pregnancies. The outcome is often unfavorable when it is presented in the second trimester and, without prophylaxis, recurrence in future pregnancies is close to 100%.^4^ The main factors contributing to occurrence or recurrence of TTP during pregnancy are a hypercoagulable state and progressive deficiency of ADAMTS13 over the course of gestation.^12^ Fibrinogen, factor VIII and von Willebrand factor levels have been found to increase up to threefold during pregnancy.^12^^,^^13^ The physiological increase in von Willebrand factor concentration seems to be directly related to the decrease in ADAMTS13 activity. Thus, women with congenital ADAMTS13 deficiency may become severely disabled during pregnancy.^12^

Relapses, defined as acute events of TTP occurring 30 days after remission, are seen in 20 to 50% of cases. They are more common in patients with low ADAMTS13 activity or with autoantibodies after remission.^14^

Differentiation between congenital and acquired TTP is needed, through measurement of ADAMTS13 activity and autoantibodies, since use of immunosuppressive therapy is critical in cases of acquired TTP but is unnecessary in cases of congenital TTP.^8^ The largest prospective study on pregnancy-associated TTP evaluated 47 women.^8^ Fetal loss occurred in 42% of the congenital TTP cases before the diagnoses, but without further losses in subsequent pregnancies if proper management was instituted. Most cases of TTP occurred in the third trimester, and only 15% of the cases occurred before the 20^th^ week. In all pregnancies in which a diagnosis of TTP was made, labor was induced at the 38^th^ week, because of the increased risk of complications that is observed in pregnancies that continue to term and beyond. That study concluded that pregnancy is a precipitating factor for TTP; that there is a risk of relapse in further pregnancies; and that, in pregnancies, late-onset congenital TTP occurs more often than acquired TTP. Treatment with plasma therapy and antithrombotic agents in further pregnancies has been found to result in improvement of fetal growth and placental histology, without fetal loss.^8^

A French study assessed 42 women who had had a first episode of TTP during pregnancy or postpartum.^4^ The rate of live births was only 31% and was associated with the time of onset of this condition: 96% of those in whom it occurred during the first and second trimester presented abortion or fetal death, compared with 17% among those who had the disease during the third trimester. Only two patients attained remission without plasma therapy, and both of them had congenital TTP.^4^

In the case reported here, the patient had four episodes of TPP: two before pregnancy (at the ages of 9 and 16 years), and two during pregnancy, both in the second trimester of gestation. During the first and third episodes of TTP, spontaneous remission (i.e. without any plasma therapy) was observed and, as far as we know, this is the only case reported in the literature. The diagnoses was suspected at the time of the second hospitalization and, if the ADAMTS13 activity level had been investigated, this would have had a great impact on the patient’s life and pregnancy outcome, since the diagnosis would have been known and she would probably have had different management.

It can be seen that suspicion of this disease is impaired through persistence of the “classical pentad” concept and the difficulty in characterizing signs and symptoms as part of a single syndrome. The differential diagnoses of microangiopathic hemolytic anemia and thrombocytopenia during pregnancy are summarized in Table 4.

**Table 4.**
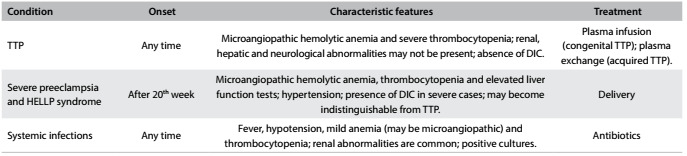
Differential diagnoses of microangiopathic anemia and thrombocytopenia during pregnancy^3^^,^^8^^,^^9^^,^^14^^,^^15^

TTP = thrombotic thrombocytopenic purpura; DIC = disseminated intravascular coagulation; HELLP = hemolysis, elevated liver-enzyme levels and low platelets.

The hemoglobinuria seen in our patient’s previous manifestations and the onset of paresthesia during the current case reflect the renal and neurological impairments of the disease that occur in severe cases. The presence of “left shift” leukocytosis, elevated inflammatory markers, leukocyturia and hematuria are often confused with infectious condition,^15^ which should always be a differential diagnosis and should be ruled out through cultures.

It is known that systemic infections may mimic TTP.^15^ From analysis on 415 consecutive patients in the Oklahoma TTP Registry who were initially diagnosed with TTP, two distinct groups were identified: 25 patients with measured ADAMTS13 activity whose symptoms were later attributed to systemic infections; and 62 patients with ADAMTS13 activity < 10% whose symptoms remained assigned to TTP. In the “systemic infections” group, more cases with fever, coma and the classical TTP pentad were identified, along with more lethal cases. In the TTP group, 31% did not show any neurological abnormalities, but the presence of relapses and focal deficits was higher and the average platelet and hematocrit levels were lower than in the first group. This study revealed that the classical clinical pentad is rare among cases of TTP and is much more common in cases of sepsis. Nevertheless, it still has great influence on many physicians, since there is usually a belief that if more features of the pentad are present, the diagnosis of TTP becomes more likely.^15^

Since no anti-ADAMTS13 autoantibodies are present in cases of congenital TTP, treatment of acute cases by means of plasma exchange or plasma infusion alone is appropriate.^3^^,^^5^^,^^14^ After remission of acute symptoms has been achieved, the treatment should be individualized according each patient’s phenotype. Some patients may require monthly plasma infusions to restore ADAMTS13 and prevent symptomatic episodes, while others only require prophylactic therapy under risky conditions such as surgery, infections, pregnancies and presentation of thrombocytopenia.^3^^,^^5^ A study involving four European TTP registries found that low residual ADAMTS13 activity was associated with early manifestations requiring plasma therapy and with higher frequency of recurrences.^16^ Since congenital TTP is hereditary, evaluation of ADAMTS13 activity among close relatives should be considered.^14^

Blood transfusions and folic acid supplementation are indicated during hemolysis, especially if there is cardiac impairment.^5^^,^^14^ However, platelet transfusions are relatively contraindicated and are only acceptable when there is life-threatening bleeding.^5^^,^^14^ Patients with TTP who receive platelet transfusions have a higher chance of arterial thrombosis (adjusted odds ratio, adjOR = 5.8; 95% confidence interval, CI = 1.3-26.6) and myocardial infarction (adjOR = 2.0; 95% CI = 1.2-3.3), and higher mortality (adjOR = 2.0; 95% CI = 1.3-3.0).^17^

In managing congenital TTP in subsequent pregnancies, low doses of acetylsalicylic acid in association with regular plasma infusion are recommended as soon the pregnancy has been confirmed, starting at a dose of 10 ml/kg every two weeks, and changing to once a week after the 20^th^ week.^8^ If the patient is thrombocyto-penic, the plasma dose may be increased to 15 ml/kg. It is recommended that delivery should take place between the 36^th^ and 38^th^ week because this has been proven to reduce fetal loss and relapses.^8^

## CONCLUSION

This report reveals the importance of suspicion of TTP in the presence of microangiopathic hemolytic anemia and thrombocytopenia that do not have any apparent cause. Waiting for the classical pentad to appear before making the suspected diagnosis and introducing treatment is unwise and can seal the outcome. Because of the risks involved in plasmapheresis, presence of systemic infection mimicking TTP needs to be ruled out.

To date, the present case provides the only description of congenital TTP with two spontaneous remissions in the literature. Spontaneous remissions may reflect a milder phenotype of TTP. Obtaining clarifications from patients regarding their condition is essential for identifying the factors that may precipitate worsening of disease and for managing possible recurrences.

## References

[1] George JN (2006). Clinical practice. Thrombotic thrombocytopenic purpura. N Engl J Med.

[2] George JN (2010). How I treat patients with thrombotic thrombocytopenic purpura: 2010. Blood.

[3] George JN, Nester CM (2014). Syndromes of thrombotic microangiopathy. N Engl J Med.

[4] Moatti-Cohen M, Garrec C, Wolf M (2012). Unexpected frequency of Upshaw-Schulman syndrome in pregnancy-onset thrombotic thrombocytopenic purpura. Blood.

[5] Blombery P, Scully M (2014). Management of thrombotic thrombocytopenic purpura: current perspectives. J Blood Med.

[6] Furlan M, Robles R, Galbusera M (1998). von Willebrand factor-cleaving protease in thrombotic thrombocytopenic purpura and the hemolytic-uremic syndrome. N Engl J Med.

[7] Levy GG, Nichols WC, Lian EC (2001). Mutations in a member of the ADAMTS gene family cause thrombotic thrombocytopenic purpura. Nature.

[8] Scully M, Thomas M, Underwood M (2014). Thrombotic thrombocytopenic purpura and pregnancy: presentation, management, and subsequent pregnancy outcomes. Blood.

[9] McMinn JR, George JN (2001). Evaluation of women with clinically suspected thrombotic thrombocytopenic purpura-hemolytic uremic syndrome during pregnancy. J Clin Apher.

[10] Amorosi EL, Ultmann JE (1966). Thrombotic thrombocytopenic purpura: report of 16 cases and review of the literature. Medicine.

[11] Allford SL, Hunt BJ, Rose P, Machin SJ, Haemostasis and Thrombosis Task Force, British Committee for Standards in Haematology (2003). Guidelines on the diagnosis and management of the thrombotic microangiopathic haemolytic anaemias. Br J Haematol.

[12] George JN (2003). The association of pregnancy with thrombotic thrombocytopenic purpura-hemolytic uremic syndrome. Curr Opin Hematol.

[13] Stirling Y, Woolf L, North WR, Seghatchian MJ, Meade TW (1984). Haemostasis in normal pregnancy. Thromb Haemost.

[14] Scully M, Hunt BJ, Benjamin S (2012). Guidelines on the diagnosis and management of thrombotic thrombocytopenic purpura and other thrombotic microangiopathies. Br J Haematol.

[15] Booth KK, Terrell DR, Vesely SK, George JN (2011). Systemic infections mimicking thrombotic thrombocytopenic purpura. Am J Hematol.

[16] Lotta LA, Wu HM, Mackie IJ (2012). Residual plasmatic activity of ADAMTS13 is correlated with phenotype severity in congenital thrombotic thrombocytopenic purpura. Blood.

[17] Goel R, Ness PM, Takemoto CM (2015). Platelet transfusions in platelet consumptive disorders are associated with arterial thrombosis and in-hospital mortality. Blood.

